# Future clinical medical physics division should have fewer medical physicists and more medical physics assistants

**DOI:** 10.1002/acm2.14592

**Published:** 2024-12-02

**Authors:** Minsun Kim, Hyejoo Kang, Yi Rong

**Affiliations:** ^1^ Radiation Oncology University of Washington Seattle Washington USA; ^2^ Radiation Oncology Stritch School of Medicine Loyola University Chicago Maywood Illinois USA; ^3^ Radiation Oncology Mayo Clinic Arizona Phoenix Arizona USA

**Keywords:** medical physics assistant, medical physics workforce, qualified medical physicist

## 1 INTRODUCTION

Medical physics is a specialized branch of applied physics that plays a critical role in healthcare, particularly in the diagnosis and treatment of disease.^1^ Medical physicists contribute directly to patient care through a range of responsibilities, such as providing clinical services, routine evaluations of equipment performance, performing quality and safety inspections, and taking on roles in administrative, education, informatic data services.^1^, ^2^ Among its many subfields, therapeutic medical physics, which focuses on applying principles of physics in radiotherapy, stands out as one of the most prominent professional service within the branch. As the technology advances in the field of radiotherapy, the role of qualified medical physicist (QMP) has evolved significantly. Tasks once considered complex, such as quality assurances for equipment performance, are now routine. Whereas the scope of responsibilities has expanded to include the integration of novel technologies and development of clinical workflows for emerging treatment methods. This evolution in practice has given rise to newer opportunities including emergence of sub‐profession, medical physics assistants (MPAs), which is designed to alleviate some of the routine workloads that traditionally lie on QMPs. As our profession continues to explore the roles and responsibilities of the emerging sub‐profession of MPA, the shortage of medical physics professionals is intensifying.^1^, ^2^, ^3^ This situation makes it crucial to evaluate how the field of Medical Physics needs to adapt. We are at a critical juncture, deciding how much to rely on MPAs and what the future workforce will look like. In this edition of debate article, we have Dr Minsun Kim and Dr Hyejoo Kang provide contrasting perspectives on the proposition “Future clinical medical physics division should have fewer medical physicists and more medical physics assistants.”

Dr Minsun Kim received her PhD degree in Applied Mathematics from the University of Washington (UW). Currently, she is a Professor at UW and serves as the Clinical Physics Lead at the UW Medical Center. Her research focuses on the clinical outcome driven radiation treatment planning and her clinical interests include adaptive radiotherapy and automation to improve patient safety and workflow efficiency. She is actively involved in the professional activities of the Association of Physicists in Medicine, The Radiosurgery Society, and American Board of Radiology. She also serves as the Physics Section Editor for Practical Radiation Oncology.

Dr Hyejoo Kang received her PhD from Rutgers University in high‐energy experimental physics and conducted postdoctoral research on a neutrino experiment at Stanford University. She completed her research fellowship in medical physics at Memorial Sloan‐Kettering Cancer Center (MSKCC) in 2010. She has held research and clinical positions at The University of Chicago and Northwestern Medicine. She is currently an Associate Professor in the Department of Radiation Oncology and serves as the Associate Director of Medical Physics at Loyola University Chicago.

## 2 OPENING STATEMENT

### 2.1 For the proposition: Minsun Kim, PhD

The traditional role of medical physicists was often limited to commissioning and maintaining equipment in the clinical department. However, the role of the medical physicist has significantly expanded in the past decade. Medical physicists bring unique expertise in medicine with a strong foundation in physics, engineering, and other quantitative disciplines, to patient care. This multidisciplinary expertise helps bridge the gap between advanced technologies and complex clinic environments to ensure safe implementation and enhance patient care, making their roles multi‐faceted in the evolving landscape of healthcare. Medical Physics 3.0 discusses the broadened and diversified areas where medical physicists can and should contribute to patient care. These new roles leverage the unique expertise and strengths that they possess as both scientists and medical professionals.^4^ Routine responsibilities of medical physicists currently include, but are not limited to, a wide range of clinical practices such as managing equipment, implementing safety protocols, and performing patient‐directed procedures, as well as conducting research and clinical development, and educating the next generation of medical physicists and other related medical professionals. I have no doubt that these roles will continue to evolve, and that new horizons will emerge as the field advances.

Various tasks that medical physicists currently perform require different levels of technical expertise and direct attention from medical physicists. For example, developing novel technologies and implementing newly available procedures into clinical practice does not require the same level of direct involvement from medical physicists as performing well‐established, routine QA procedures on existing equipment. Assigning medical physicists to tasks that do not require their direct, real‐time involvement wastes their specialized skills and increases costs unnecessarily within the healthcare system. A more effective approach is to delegate these tasks to appropriately trained MPAs under direct or indirect supervision of medical physicists, depending on the nature of the delegated tasks. This approach shifts the workload, resulting in fewer medical physicists and more MPAs handling the same amount of medical physics work currently performed within the clinical department.

Employing medical physicists to perform complex and specialized activities that require advanced training and competencies as a QMP can not only enhance overall efficiency in the healthcare system but also offer a fulfilling and rewarding career path for talented professionals, with the potential for higher income. By focusing on tasks that truly require their expertise and unique skill sets, medical physicists can achieve greater career satisfaction in an environment that empowers them to reach their full potential. This approach will attract more motivated and engaged professionals to the field of medical physics, ultimately benefiting both the healthcare system and the patients it serves.

Additionally, the MPA program not only addresses the operational needs of healthcare institutions but also offers valuable learning opportunities and financial support for students and those interested in the field of medical physics. Through the MPA program, trainees gain hands‐on clinical experience and insights through paid positions, which enhances their practical knowledge and skills in a real‐world setting. Unlike programs that primarily focus on training, such as unpaid internships or practicums, the MPA program emphasizes task‐driven hands‐on experience and values individuals’ labor with proper monetary compensation. This compensation helps alleviate the significant financial burden associated with pursuing a career in medical physics. The average tuition and fees for master's programs in medical physics exceeded $30 000 per year in 2023,^5^ representing a substantial obstacle for many talented individuals. By offering paid positions, the MPA program provides an alternative pathway for aspiring medical physicists to gain essential experience without incurring excessive debt, thereby enhancing accessibility. This financial support not only broadens access to the field but also helps healthcare institutions attract and retain diverse, capable individuals who might otherwise be deterred by the high cost of education.

To safely integrate MPAs into routine clinical practice, it is crucial to clearly define their qualifications and scope of work, as well as establish the level of supervision required from medical physicists.^6^ Setting these guidelines ensures that MPAs contribute effectively to the healthcare system without compromising care quality delivered to patients. This structured approach allows medical physicists to focus on more complex and critical tasks that require their expertise, while overseeing MPAs who handle tasks that do not necessitate their direct involvement. Allocating more MPA positions with fewer medical physicists optimizes departmental resources, and enhances overall efficiency within the healthcare system, allowing medical physicists to dedicate their time and skills to areas where they can have the greatest impact.

### 2.2 Against the proposition: Hyejoo Kang, PhD

In recent years, the medical physics community has faced a workforce shortage due to several factors, including the impact of COVID‐19 and anticipated retirements. The community recognizes the need for alternative solutions to address this issue, such as expanding residency programs and employing MPAs as professionally trained quality assurance staff. It is known that Commission on Accreditation of Medical Physics Education Programs (CAMPEP)‐accredited residency programs may not produce enough QMPs to meet both current and future demand.^1^, ^3^, ^7^ The recent trend in medical physics involves employing MPAs to perform more routine and less complex clinical tasks, such as patient‐specific QA, monthly QA, and basic treatment planning. MPAs in general require less extensive training over a relatively short period, compared to the amount of training for a QMP.

However, this trend might pose risks to our profession. Hospital administrators may perceive that all QMPs can be replaced by MPAs, unless differentiations in the roles and responsibilities of MPAs and QMPs are clearly defined and periodically presented to them. Given that MPAs have limited training and knowledge in our field, an increase in their numbers, if not accompanied by adequate supervision, may lead to a decline in the quality of medical physics training and potentially compromise patient care. Currently, there is a lack of systematic guidelines on the training and clinical responsibilities of MPAs, leading to variability in their roles across institutions. Moreover, there is an increasing potential for scenarios where MPAs outnumber QMPs in clinical settings. This could create a precarious situation in which hospital senior management might view this imbalance as acceptable, potentially leading to the expansion of this practice.

Additionally, MPAs still require a certain level of training and clear guidance on when to defer to QMPs for decision‐making. Recent advancements in medical physics, including the integration of multiple imaging modalities and adaptive radiation therapy, have increased the complexity of clinical practice. Consequently, the demand for QMPs has grown, and there may be fewer “routine” tasks that MPAs can perform independently. This evolution in our field underscores the need for the American Association of Physicists in Medicine (AAPM) to establish further guidelines for MPA training models, distinct from those for QMP training.

Ideally, our efforts should focus on training more residents through structured programs led by experienced QMPs. These programs provide rigorous clinical and didactic training, equipping residents with the skills to think critically, apply fundamental physics principles in clinical decision‐making and develop professionalism.^8^, ^9^ It is crucial for residents to be actively involved in clinical processes and activities, and to receive proper evaluation through a structured feedback process. The current MPA training does not require these essential steps, making it difficult to assess their competency in clinical settings.^6^ Therefore, it would be discreet to first clearly define the scope of MPA's responsibilities and establish comprehensive MPA training, rather than hiring them as a temporary workforce in clinic.

Given those consideration, it seems more prudent to first expand well‐established residency programs, which are supported by organizations such as AAPM, CAMPEP, and American Board of Radiology (ABR), to train the next generation before branching out into additional training programs.

## 3 REBUTTAL

### 3.1 For the proposition: Minsun Kim, PhD

It is our responsibility to elucidate to decision‐makers within the healthcare system the role of QMPs and their impact on patient care. The role of QMP and their competencies are not determined by the perceptions of hospital administrators but are established by the medical physicists themselves and the professional societies representing them. I concur with Dr Kang that we should not let the number of QMPs decrease to a level where the quality of patient care is compromised, as it is everyone's shared mission to best serve patients, regardless of their specific role within the healthcare system. The focus should be on the efficient use of appropriate resources, ensuring that tasks are performed safely and effectively by those best suited professionals.

As Dr Kang correctly pointed out, shifting some of the medical physics workload to MPAs require clear guidelines and an evaluation of its impact on patient care. This need is well recognized by AAPM, and MPPG 7a was published to clarify the role of QMPs and MPAs. It clearly states that the QMP is solely responsible for medical physics practice in the department, and the work of MPAs must be directed by a QMP. Therefore, in line with professional society recommendations and standards, medical physicists should determine the scope of MPA work and the appropriate number of MPAs needed to safely manage all types of medical physics tasks, which are unique to each department.

I fully support Dr Kang's point that maintaining a permanent, stable workforce is better than relying on temporary staff in the clinic, as it provides consistency. In multi‐disciplinary environments where medical physicists work, building strong relationships with various professionals within the department is crucial for best serving our patients. Indeed, this is particularly challenging when using a temporary workforce. However, the tasks delegated to MPAs are often related to routine QA. These tasks typically do not require close contact with other professionals in the department, aside from the supervising medical physicists. The nature of delegated tasks can alleviate concerns often associated with temporary staff, and the residency program does not fully address these concerns, as residents can also be seen as temporary. Furthermore, as the MPA program matures, it may become an established profession for those who find it aligns with their personal or professional goals, rather than just a temporary role before transitioning to a medical physicist. MPA positions as permanent career choices may become more prevalent, offering long‐term fulfillment and stability.

As the field of medical physics rapidly evolves, new and unforeseen opportunities are emerging. Advances in technology offer medical physicists innovative ways to contribute to patient care beyond the clinical department. As the field progresses, the responsibilities of medical physicists should adapt to meet the demands of these advancements. It is time to delegate clinical tasks that are well‐established and do not require the advanced skill sets for which medical physicists are trained. This approach will help optimize resources and improve job satisfaction for both medical physicists and MPAs.

### 3.2 Against the proposition: Hyejoo Kang, PhD

Dr Kim has highlighted the critical evolution in the responsibilities of medical physicists encompassing a multidisciplinary expertise. This evolution underscores the need for advanced skills to address challenges posed by advanced technologies and complex clinic issues. I fully agree that our efforts should shifts towards enhancing our skills and experience in these areas, rather than concentrating on “routine” clinical tasks, as our roles extend beyond equipment commissioning and maintenance and checking treatment plans. It is anticipated that QMPs play a key role in safe and effective implementation of new technologies, enhancing patient care, and contributing to scientific research, education, and clinical innovation.

If we can receive assistance from properly trained MPAs while providing them with financial and educational benefits, we have a pragmatic solution for meeting the growing demands for physics support while maintaining high standards of patient care. However, training and supervising MPAs would increase the responsibilities of QMPs, adding to their already demanding duties and workload.

The proposal of delegating routine tasks to MPAs, while theoretically improving efficiency, still raises major concerns about unclear definitions of roles, particularly in clinics already facing a shortage of QMPs. In such environments, where QMPs are already overwhelmed with clinical and educational duties, they may struggle to define clear roles or ensure adequate training for MPAs. It takes a substantial amount of time and effort to train MPAs on clinical duties, and due to the temporary nature of their positions, many MPAs might leave the institution within one or two years. As a result, QMPs must repeat the training for incoming MPAs, which can become an unsustainable burden for QMPs. It seems unrealistic to expect QMPs at each institution to concretely define the roles of MPAs and provide uniform training, rather than adopting guidelines from national organizations.

While MPAs might be able to perform routine tasks, these are often limited and may not significantly reduce the workload of QMPs. Among the routine responsibilities of medical physicists, as Dr Kim mentioned, such as managing equipment, implementing safety protocols, and performing patient‐related procedures in clinic, it is unlikely that MPAs could independently perform these tasks without considerable oversight from QMPs, as these responsibilities have a significant impact on patient care. In reality, QMP may need to spend even more time and effort in training and supervising MPAs to ensure safe physics support, which could counteract the intended benefits of employing MPAs. The introduction of MPAs could increase the teaching burden on QMPs, requiring them to divide their efforts between residency programs and MPA training. This could be inefficient and counterproductive to maintaining high‐quality patient care and education for both institutions and QMPs.

I also agree with Dr Kim's point on providing financial support for students tremendously benefits the student while they are gaining clinical experience. However, not all institutions employing MPAs offer such graduate programs. For some, the MPA role might be a temporary position or a stepping‐stone toward residency applications. The variability in clinical training of MPA across institutions can lead to inconsistent training level when they enter residency programs. This could necessitate modifications to residency programs to account for prior MPA experience, potentially leading to redundancies and inefficient use of training resources. Furthermore, without standardized MPA training guidelines, new residents may enter programs with varying levels of preparedness, complicating the training process. As a result, QMPs may end up spending more time to refine the residency program to accommodate individual resident with different levels of prior training. This might add complexity that potentially QMPs might have to dedicate a substantial amount of their time.

As I stated in my opening argument, expanding residency programs appears to be the most appropriate path for acquiring the skills and experience for this future direction, given their structured frameworks and requirements from various internal and external organizations. Many residency programs are structured to keep pace with the evolving demands of our field, with rotation modules and content being regularly reviewed and updated by different committees. Additionally, the comprehensive clinical experience gained during clinical operation hours naturally equips residents with the ability to tackle complex problems and integrate new technologies into practice. Therefore, I believe that expansion of residency program is the optimal approach for training the next generation of QMPs. We should focus on training and education for our residents adopting the broaden approach in our responsibilities in addition to mastering fundamental knowledge including both traditional and emerging aspects of medical physics with adequate resources.

Dr Kim and I both agree that employing MPAs can indeed alleviate the current shortage of QMPs and ensure proper patient care if integrated with caution and standard guidelines. We concur that with proper training and well‐defined roles, having more MPAs can benefit both clinical support and their own development and financial assistance. However, as we have both noted, for this to be a sustainable solution, standardized guidelines should be established to define responsibilities and elaborate training requirements for MPAs, with input from all stakeholders including AAPM, CAMPEP, physicists, educators, and hospital administrators. However, most importantly, reducing the number of QMPs in the hospital may compromise safe integration of technology into the clinic, patient care, education, and advancements for the field of medical physics.

## AUTHOR CONTRIBUTIONS

Minsun Kim and Hyejoo Kang contributed equally to drafting the manuscript. Yi Rong contributed in manuscript drafting.

## CONFLICT OF INTEREST STATEMENT

The authors declare no conflicts of interest.
